# Loss-Chasing, Alexithymia, and Impulsivity in a Gambling Task: Alexithymia as a Precursor to Loss-Chasing Behavior When Gambling

**DOI:** 10.3389/fpsyg.2016.00003

**Published:** 2016-01-20

**Authors:** Peter A. Bibby

**Affiliations:** School of Psychology, The University of NottinghamNottingham, UK

**Keywords:** loss-chasing, alexithymia, impulsivity, gambling

## Abstract

**Objective:** To examine the relationship between loss-chasing, the propensity to continue gambling to recover from losses, alexithymia, a personality trait associated poor emotional processing and impulsivity, the tendency to act quickly without reflection or consideration of the consequences.

**Method:** Two experiments are reported (E1: *N* = 60, Males, 11; Age, 21.6 years. E2: *N* = 49, Males, 22; Age, 21.1 years). In experiment 1, two groups (low alexithymia, high alexithymia) completed the Cambridge Gambling Task (CGT). Loss-chasing behavior was investigated. In experiment 2, both alexithymia (low, high) and impulsivity (low, high) were examined also using the CGT. A further change was the order of bet proportion from ascending to descending.

**Results:** Experiment 1 shows loss-chasing behavior in participants high in alexithymia but not those low in alexithymia ($\eta_{p}^{2}=0.09$). Experiment 2 shows loss-chasing behavior in participants both low and high in alexithymia but it was greater for participants high in alexithymia ($\eta_{p}^{2}$ = 0.09). The effect of impulsivity was not statistically significant ($\eta_{p}^{2}$ = 0.01). Loss-chasing behavior was correlated with the emotional facets of alexithymia but not the cognitive facet.

**Conclusions:** Alexithymia is a precursor to loss-chasing when gambling and loss-chasing reflects the cognitive and emotional aspects of gambling. Specifically, the tendency to loss-chase depends on the need to recoup previous losses and failure to process the emotional consequences of those losses.

## Introduction

For the vast majority of people, gambling is a form of entertainment, occasionally indulged at a minimum cost (e.g., lottery players). The British Gambling Prevalence Survey 2010 (Wardle et al., 2011) surveyed 7756 individuals and found that in the last year the number of times people played the National Lottery was 3.2, slot machines was 5.6, and horse races was 5.0. However, for a small group of individuals gambling is a serious problem with negative consequences for the individual, their family, and society as a whole. The percentage of the population who are problem gamblers varies from 0.3 (Sweden) to 5.0% (Hong Kong) with the UK near the median at 0.9% (Wardle et al., 2011). Though these percentages seem small the number of problem gamblers is large. The UK estimate is between a quarter and half a million people (Wardle et al., 2011). Given the prevalence of problem gambling it is important to examine the mechanisms both distal (e.g., personality traits) and proximal (e.g., loss-chasing behavior) that lead to problem gambling. This paper examines two personality traits, alexithymia and impulsivity, and a key feature of problem gambling, loss-chasing behavior.

### Loss-chasing and personality

The DSM-IV (Diagnostic and Statistical Manual of the American Psychiatric Association, Fourth Edition) criteria for problem gambling includes “chasing” one's losses, that is continuing to gamble, often with increasing bet size, to recover from losses. This phenomenon is common among problem gamblers and may be the most significant step on the road to problem gambling (Lesieur, 1979; Dickerson et al., 1987; Corless and Dickerson, 1989; O'Connor and Dickerson, 2003). Toce-Gerstein et al. (2003) found that more than 75% of problem gamblers reported chasing losses and 59.6% of all gamblers chased. They also found that chasing losses occurred even when other commonly cited indicators of problem gambling did not.

Breen and Zuckerman (1999) point out that the common view of chasing involves returning on a later day. However, continuing to gamble maladaptively by chasing within a single gambling session is highly likely to be a contributing factor in the development of the between session chasing behavior. It is this kind of “chasing” that is also the focus of the current paper.

Breen and Zuckerman (1999) examined within session “chasing.” In their experiment, chasers were defined as those participants who continued to play a gambling game until they had lost all their money. The game was designed so that losing all the money was an inevitable consequence on continuing to play. Out of 203 participants 70 (34%) were categorized as chasers. The only personality measure that showed a significant difference between players who chased and those who did not was impulsive sensation seeking. They argued that this reflects a difference in sensitivity to rewards and punishments (c.f., Gray and McNaughton, 2000) with punishments being relatively ineffective in reducing loss-chasing.

A study by Linnet et al. (2006) examined problem gamblers and non-problem gamblers in the context of the Iowa Gambling Task (IGT; Bechara et al., 1994). They found that problem gamblers showed evidence of more loss-chasing than non-problem gamblers. Problem gamblers, they argued do not notice their chasing behavior. This is consistent with the idea of hyposensitivity to losses. You may not notice that you are throwing good money after bad if you have not noticed it is bad.

Kim and Lee (2011) examined the influence of the Behavioral Approach System and Behavioral Inhibition System on decision making in a simple gambling task. This task allowed Kim and Lee (2011) to examine behavior after wins and losses. They found that the combination of high behavioral approach and low behavior inhibition was associated with more risky decisions after a win but the combination of low behavioral approach and high behavioral inhibition was related to fewer non-risky decisions after losses. They hypothesized that the experience of losses facilitates the inhibitory behavior, suggesting an increased sensitivity to losses. Kim and Lee (2011) suggest that further research is required to examine the relationship between personality traits and loss-chasing behavior.

### Alexithymia and problem gambling

Alexithmyia is a stable personality trait associated with the processing of emotional information (Taylor et al., 1997). The key features of alexithymia have been identified as difficulty identifying feelings (DIF), difficulty describing feelings (DDF), and externally oriented thinking (EOT; Parker et al., 1993, 2008; Bagby et al., 1994, 2007, 2009). Essentially, a person high in alexithymia finds making sense of their own and other people's emotions difficult. As a consequence they tend to focus on external rather than internal causes for behavior.

Alexithymia is related to problem gambling (Lumley and Roby, 1995; Parker et al., 2005; Toplak et al., 2007; Bonnaire et al., 2009; Ferguson et al., 2009; Mitrovic and Brown, 2009). Lumley and Roby (1995) used the South Oaks Gambling Screen (SOGS; Lesieur and Blume, 1987) and Toronto Alexithymia Scale (TAS-20; Taylor et al., 1985) to examine the relationship between alexithymia and problem gambling. Of the 1100 American university students, 3.1% were identified as problem gamblers using the SOGS criteria. Of these, 34% were identified as alexithymic (a high degree of alexithymia) whereas only 11.1% of the non-problem gamblers were so classified. Parker et al. (2005), using the revised Toronto Alexithymia Scale (TAS-20, Parker et al., 1993) and SOGS, found that 22% of the pathological gamblers were alexithymic whereas only 11% of the non-problem gamblers were alexithymic. Though Bonnaire et al. (2009) only examined pathological gamblers they found that 44% were identified as alexithymic. Parker et al. (2008) found a 10% incidence of alexithymia in a community sample (*n* = 1933) and an 11% incidence in an undergraduate sample (*n* = 1948). Thus, an incidence of 44%, as observed by Bonnaire et al. (2009) is greater than would be expected.

### Alexithymia and loss chasing

There are two areas of research that suggest that there is a link between the alexithymia and loss-chasing. First, it has been suggested that people who are high in alexithymia have difficulty processing information about losses (Ferguson et al., 2009; Bibby and Ferguson, 2011) and second, the neurological structures implicated in loss-chasing (Campbell-Meiklejohn et al., 2008) show clear differences in alexithymic and non-alexithymic individuals (Lane et al., 1998; Berthoz et al., 2002; Kano et al., 2003, 2007; Mantani et al., 2005; Moriguchi et al., 2006).

Ferguson et al. (2009) examined the behavior of people scoring low and high on the TAS-20 when completing the IGT. They found that the rate at which participants high in alexithymia learned was slower. Furthermore, alexithymic participants returned to deck B significantly more often than expected by chance, whereas non-alexithymic participants did not. Deck B has been identified as being distinct from the other decks (Lin et al., 2007). The schedule of rewards for Deck B is that on each trial (in a block of 10 trials) there is a relatively high gain and only one single, catastrophic loss. Ferguson et al. (2009) suggest that the willingness to return to deck B indicates that participants high in alexithymia are less sensitive to losses.

Bibby and Ferguson (2011) followed up this suggestion and examined the relationship between loss aversion and alexithymia. They found that alexithymia was associated with loss aversion in both a riskless and a risky task. For the riskless task, higher alexithymia was associated with a smaller endowment effect (Kahneman et al., 1990), indicating less loss aversion. For the risky task, a simple lottery, higher alexithymia was associated with a willingness to accept higher potential financial losses. This tendency to less loss aversion remained even when sex, the Big 5 personality variables and sensation seeking were statistically controlled.

Campbell-Meiklejohn et al. (2008) found loss-chasing was associated with increased activity with the ventro-medial prefrontal cortex (vmPFC) and the subgenual anterior cingulate cortex (sgACC), but the decision not to loss-chase was associated with increased activity in the dorsal anterior cingulate cortex (dACC), the ventral striatum and the anterior insula cortices. Kugel et al. (2008) found a relatively strong negative correlation between TAS-20 scores and right amygdala activation when processing emotional facial expressions. Lane et al. (1998), Berthoz et al. (2002), Kano et al. (2003) all found reduced activity in the anterior cingulate cortex for alexithymics when processing emotional information. Mantani et al. (2005) reduced activity in the posterior cingulate cortex when alexithymics were asked to imagine past and future happy, sad and neutral events. Kano et al. (2003) also found an association between alexithymia and reduced activity in the anterior insular cortex. Both Kano et al. (2003) and Moriguchi et al. (2006) found reduced activity in alexithymics when processing emotional information in the pre-frontal cortex.

Given that alexithymia is associated with problem gambling and problem gambling is associated with loss-chasing and that activity in several of the same brain regions is associated with both loss-chasing and alexithymia it seems realistic to predict a relationship between between alexithymia and loss chasing in gambling. Experiment 1 uses a simple gambling task, the Cambridge Gambling Task (CGT; Rogers et al., 1999) to test the prediction that higher alexithymia is associated with greater loss-chasing. It is predicted that participants high in alexithymia will bet more after a loss than after a win and will do so more than people low in Alexithymia.

## Experiment 1

### Method

#### Participants

Sixty undergraduate student volunteers participated in the experiment (49 female, 11 male). The average age was 21.6 years. Participants were informed that there were monetary prizes of £25, £15, and £10 to be awarded for the three highest scores. Using a cut off point of ≤ 51 on the TAS-20 (Taylor et al., 1997), 42 participants were identified as non-alexithymic (36 female, 6 male) with the remaining participants at or near caseness for alexithymia (13 female, 5 male). This equates to the finding that approximately 30% of people in the general population score 52 or above on the TAS-20 (Parker et al., 2008). The chi square test of sex by alexithymia group was not significant [χ^2^(1) = 1.532, *p* = 0.216]. The means and standard deviations of the alexithymia scores by sex and alexithymia group are shown in Table 1. As expected a 2 × 2 (sex by alexithymia) between groups analysis of showed a significant main effect of alexithymia [*F*_(1, 56)_ = 46.14, *MSe* = 23.913, *p* < 0.001, $\eta_{p}^{2}$ = 0.61] with the non-alexithymic group scoring lower than the at or near caseness group. There was no effect of sex [*F*_(1, 56)_ = 0.63, *MSe* = 23.913, *p* = 0.41, $\eta_{p}^{2}$ = 0.01]. The interaction was also not significant [*F*_(1, 56)_ = 0.10, *MSe* = 23.913, *p* = 0.75, $\eta_{p}^{2}$ < 0.01]. Given that there is no evidence that sex is related to alexithymia in this sample sex was not included in the analyses that follow in the Results section.

**Table 1:** **Mean (and standard deviations) of the TAS-20 scores for sex by alexithymia group**.

	**Female**	**Male**
Non-alexithymic	41.47 (5.11)	43.33 (5.01)
At or near caseness	58.00 (4.02)	58.80 (5.12)

#### Toronto alexithymia scale (TAS-20)

Alexithymia was assessed using the 20-item Toronto Alexithymia Scale (TAS-20; Bagby et al., 1994). The TAS-20 is a self-report measure of alexithymia that includes 20 items that divide into three factors; DIF, difficulties in describing feelings (DDF), and EOT. Each item is responded to on a 1–5 point Likert Type scale with 1 representing strong disagreement and 5 representing strong agreement with higher total scores indicating higher alexithymia. Taylor et al. (1997) suggest a cut-off point of 52 on the scale as at or near caseness for a diagnosis of alexithymia.

#### Cambridge gambling task

**Figure 1:**
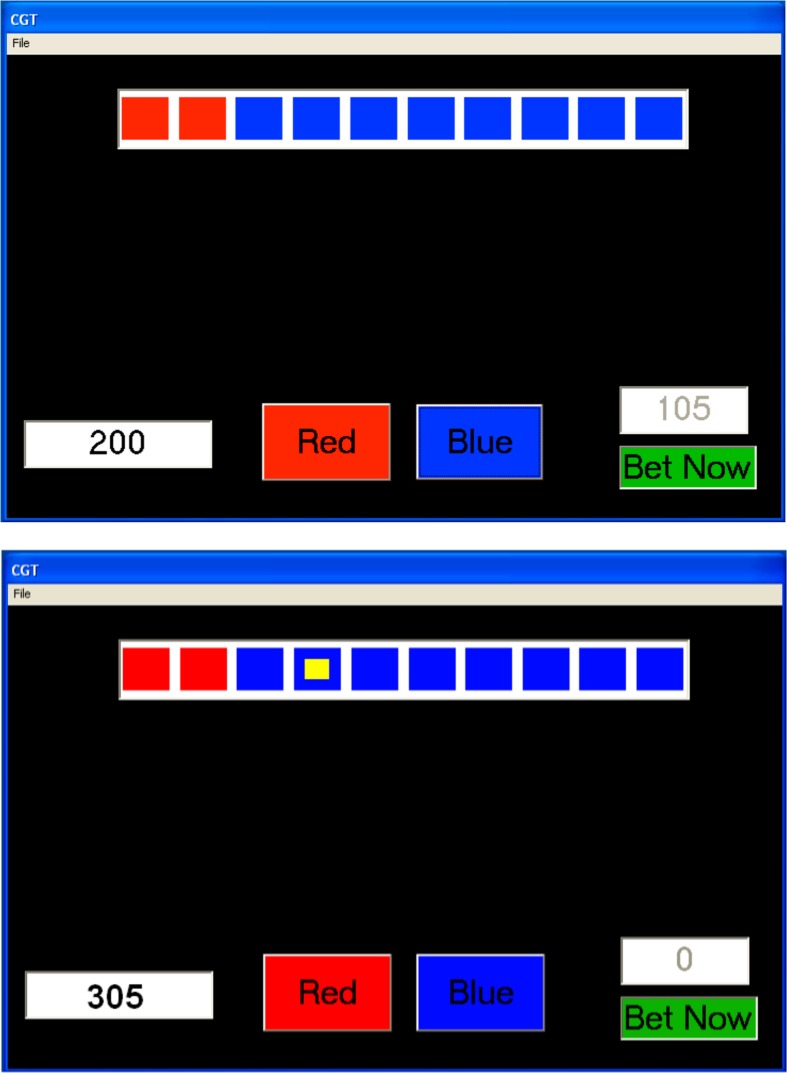
**The Cambridge Gambling Task program windows immediately before a bet is made (top, BLUE selected) and immediately after a bet is made (bottom)**.

An adapted version of the CGT (Rogers et al., 1999; see Figure 1) was used. In this task 10 boxes are displayed at the top of the program window. Ordered from left to right a number of boxes are shown in red or blue. On each trial the number of red boxes randomly varies between one and nine with an equal likelihood of each of the nine outcomes. The participant was told that the computer had randomly hidden a yellow token inside one of the boxes. The actual placement of the yellow token was randomly varied on each trial between boxes 1 and 10 with an equal likelihood of each box. The participant was told that they had to decide which color box, either RED or BLUE, the yellow token was hidden inside. She/he made this decision by clicking on the buttons shown at the bottom of the screen (RED or BLUE). To the left of these buttons is a field that shows the amount of points available to bet. This stake was incremented with any wins and decremented by losses. If at any time the stake fell below 200 points it was automatically increased back to 200 points and the computer informed participants that this had happened.

To the right of the bottom of the program window is another field which shows the actual bet to be made. The value shown in this field increments upwards in 10% steps at 1 s intervals until 90% of the available stake is shown. If a participant waited too long at the 90% step the computer cycled back round to 10% and incremented upwards in 10% steps again. The actual amount shown had a randomly generated amount of positive/negative error (up to 10% of the 10% step) to mask that it was in 10% steps. Below this field is a button that states “BET NOW.” When a participant clicked on this button the amount displayed in the field above was bet. Immediately following this selection the position of the yellow token was displayed in one of the boxes at the top of the program window and 2 s later, a message was shown stating either “You Win!” or “You Lose!” If the participant selected the correct color (e.g., she/he selected RED and the yellow ball was in a red box) then the available stake was increased by the amount bet. If the selection was incorrect (e.g., she/he selected RED but the yellow ball was in blue box) then the available stake was decreased by the amount bet. The example in the top of Figure 1 shows that the participant bet 105 out of 200 points having selected BLUE. The bottom of Figure 1 shows that the yellow ball was in a blue box and the 105 points won where transferred to the field indicating the available stake.

Each participant was told that there was a monetary reward associated with their performance. Further, that the participants scoring the top three scores in the game would win prizes of £25, £15, and £10, respectively. After 10 practice trials (with no betting involved), the participant played the gambling task (with betting) for 100 trials. Once the trials were completed the participant was debriefed and thanked for her/his participation. When all the data had been collected the participants who scored one of the top three scores where contacted and given the appropriate prize money.

This study was carried out in accordance with the recommendations of the School of Psychology, University of Nottingham Ethics committee with written informed consent from all participants. All participants gave written informed consent in accordance with the Declaration of Helsinki.

### Results

Overall, the total number of points scores across the participants was positively skewed thus the total number of points was logarithm (base 10) transformed. A *t*-test to examine whether the non-alexithymic (mean = 8.60 × 10^7^) and alexithymic (mean = 1.03 × 10^8^) participants differed in total score was not significant (*t*_58_ = −0.16, *p* = 0.87, *d* = 0.32).

To test whether participants gambled a proportion of the available stake in line with the likelihood of winning on any particular trial a 2 × 5 mixed analysis of variance was conducted on the proportion bet. The means and standard errors are found in Table 2. The first variable was alexithymia group (between groups) and the second was probability of winning (within groups) on each trial^1^ (*p* = 0.5, *p* = 0.6, *p* = 0.7, *p* = 0.8, *p* = 0.9). The effect of alexithymia was not significant [F_(1, 58)_ < 0.01, *MSe* = 0.08, *p* = 0.96, $\eta_{p}^{2}$ < 0.01]. The effect of probability of winning was significant [*F*_(1.58, 91.35)_ = 55.49, *MSe* = 0.02, *p* < 0.01, $\eta_{p}^{2}$ = 0.49]. The interaction between alexithymia and probability of winning was also not significant [*F*_(1.58, 91.35)_ = 0.15, *MSe* = 0.02, *p* = 0.96, $\eta_{p}^{2}$ < 0.01].

**Table 2:** **Means (and standard errors) of the proportion bet for the alexithymia groups by the probability of winning**.

	**Probability of winning**				
	***p* = 0.5**	***p* = 0.6**	***p* = 0.7**	***p* = 0.8**	***p* = 0.9**
Non-alexithymic	0.13 (0.02)	0.18 (0.02)	0.22 (0.02)	0.28 (0.02)	0.37 (0.03)
At or near caseness	0.15 (0.03)	0.18 (0.03)	0.23 (0.03)	0.28 (0.04)	0.36 (0.05)

To test whether participants engaged in loss-chasing a 2 (alexithymia: high vs. low) × 2 (previous trial: win vs. loss) × 5 (p of winning on each trail: *p* = 0.5, *p* = 0.6, *p* = 0.7, *p* = 0.8, *p* = 0.9) mixed analysis of variance was conducted on the proportion bet. The means and standard errors are shown in Table 3. The number of participants included in this analysis fell to 57. This was because three participants had missing values. This is in part due to the random generation of the trials interacting with participant choices such that for these participants a specific combination of won or lost at the five levels of winning probability did not occur.

**Table 3:** **Means (and standard errors) of the proportion bet for the alexithymia groups by outcome of the previous trial and probability of winning on the current trial**.

**Alexithymia**	**Previous trial**	**Probability of winning**				
		***p* = 0.5**	***p* = 0.6**	***p* = 0.7**	***p* = 0.8**	***p* = 0.9**
Non-alexithymia	Won	0.12 (0.02)	0.17 (0.02)	0.22 (0.02)	0.27 (0.03)	0.35 (0.03)
	Lost	0.14 (0.03)	0.18 (0.02)	0.22 (0.03)	0.29 (0.03)	0.36 (0.04)
At or near caseness	Won	0.13 (0.03)	0.19 (0.03)	0.20 (0.03)	0.27 (0.04)	0.34 (0.05)
	Lost	0.19 (0.04)	0.19 (0.03)	0.27 (0.04)	0.28 (0.04)	0.42 (0.05)

The main effects of previous trial [*F*_(1, 55)_ = 19.21, *MSe* < 0.01, *p* = < 0.01, $\eta_{p}^{2}$ < 0.26] and probability of winning [*F*_(1.83, 100.52)_ = 46.14, *MSe* = 0.04, *p* < 0.01, $\eta_{p}^{2}$ = 0.46] were both significant but the main effect of alexithymia was not [*F*_(1, 55)_ = 0.18, *MSe* = 0.17, *p* = 0.68, $\eta_{p}^{2}$ < 0.01]. Overall, participants bet a greater proportion of the available stake after they had lost on a previous trial (mean = 0.26) than won (mean = 0.23). Overall as the probability of winning increased participants bet a larger proportion of the available stake (*p*_0.5_ = 0.15, *p*_0.6_ = 0.18, *p*_0.7_ = 0.23, *p*_0.8_ = 0.28, *p*_0.9_ = 0.37).

**Figure 2:**
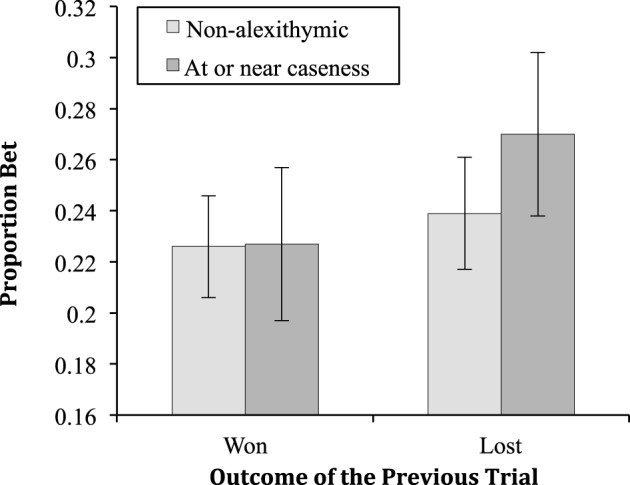
**The proportion bet (means and standard errors) for the alexithymia groups having won or lost on the previous trial**.

The two-way interaction between alexithymia and previous trial was significant [*F*_(1, 55)_ = 5.50, *MSe* < 0.01, *p* = 0.02, $\eta_{p}^{2}=0.09$; see Figure 2]. Neither the two-way interaction between alexithymia and probability of winning [*F*_(1.83, 100.52)_ = 46.14, *MSe* = 0.04, *p* = 0.77, $\eta_{p}^{2}$ < 0.01] nor that between previous trial and probability of winning [*F*_(3.38, 185.96)_ = 1.55, *MSe* < 0.01, *p* = 0.20, $\eta_{p}^{2}$ = 0.03] were significant. Finally, the three-way interaction between alexithymia, previous trial and probability of winning was not significant [*F*_(3.38, 185.96)_ = 2.32, *MSe* < 0.01, *p* = 0.06, $\eta_{p}^{2}$ = 0.040].

The simple effects of the two-way interaction between alexithymia and previous trial showed that there was no significant difference in proportion bet between the alexithymia groups when the previous trial was won (*p* = 0.99) or lost (*p* = 0.44). For the non-alexithymia group there was no difference between the proportion bet for wins and losses (*p* = 0.08), however, there was for the group at or near caseness (*p* < 0.01). For the three-way interaction the key finding of the simple effects analysis is that for the non-alexithymic group there are no significant differences in the proportion bet whether the previous trial was a win or loss at any probability of winning. On the other hand, for the at or near caseness group there were significant differences in the proportion bet after a win or a loss at the *p* = 0.5 (*p* = 0.02), *p* = 0.7 (*p* < 0.01), and *p* = 0.9 (*p* < 0.01) levels of winning probability.

### Discussion

The results showed both low and high alexithymia participants bet a proportion of the available stake that increased linearly with the probability of winning. It seems likely then that both groups understood the nature of the task and behaved in a rational manner with respect to the task—bet more when the odds of winning are greater. With respect to loss-chasing the important finding is that those low in alexithymia showed no evidence of betting more after a loss (22.6% of the available stake) than after a win (23.9%). In other words, the amount they bet overall was apparently independent of their prior experience of winning or losing. However, participants high in alexithymia (at or near caseness) showed a loss-chasing effect. After a win they bet 22.7%, an almost identical proportion as the non-alexithymic participants. However, they bet significantly more, 27.0%, after a loss. In other words, they chased their losses.

Unlike previous research, there is no evidence that those low in alexithymia chased losses. This could reflect the fact that these participants on average rarely bet more than 40% of their available stake, even though the probability of winning could be as high as *p* = 0.9. Thus, a catastrophic loss, with the ensuing need to regain the points as quickly as possible to maximize the overall score, generally did not happen. It is possible that if the amount lost was increased then participants low in alexithymia may loss-chase (in terms of the amount bet). Similarly, even though there is evidence of loss-chasing for participants high in alexithymia, the effect is relatively small (*d* = 0.27). In fact, the finding that there was no significant difference between low and high alexithymia groups on the overall score (two prizes were won by low alexithymia scorers and one prize by a high alexithymia scorer) suggests that in this task, at least, loss-chasing does not lead to a spiral of further loss-chasing.

To mimic the kind of serious loss that might facilitate loss-chasing it was decided to modify the experiment such that participants were initially offered high bets, decreasing with time. This may produce the type of deep losses that would provoke further loss-chasing. However, it does introduce a further possible explanation, impulsivity. If participants are high in impulsivity they may find it difficult to wait sufficiently long to reach the lower stake bets. Impulsivity has been shown to be related to both problem gambling (Blaszczynski et al., 1997; Steel and Blaszczynski, 1998; Vitaro et al., 1999, 2004; Alessi and Petry, 2003; MacLaren et al., 2011) and alexithymia (Gustavsson et al., 2003; Zimmermann et al., 2005; Gunnarsson et al., 2008; Wickens et al., 2008; Shishido et al., 2013). Given that impulsivity is related to both, it is necessary to take into account the affect that impulsivity could have on loss-chasing.

## Experiment 2

Experiment 2 was designed to extend the findings of the first experiment. It is predicted that by increasing the likelihood of a large possible loss by increasing the initial bet value to 90% of the available stake, both low and high alexithymia participants will demonstrate loss-chasing behaviors but it will be greater for the high alexithymia participants. Furthermore, it is predicted that high impulsivity participants will be more likely to loss-chase, as they will be more likely to find themselves in the position of making larger losses. Finally, it is hypothesized that the combination of high alexithymia and high impulsivity will be particularly toxic for loss-chasing and likely to boost loss-chasing behavior.

### Method

#### Participants

Initially 176 university undergraduates were asked to complete both the TAS-20 and the BIS-11 Scales. These participants were not paid for their participants but they were asked whether if contacted later they would be willing to participate in a further experiment in which they had the opportunity to win a monetary prize. A computer program then selected randomly (using a constraint-based iterative algorithm) participants to be contacted for participation in the experiment. Four lists of 20 participants to be contacted were generated, crossing higher and lower alexithymia with higher and lower impulsivity as measured by the TAS-20 and BIS-11 scales, respectively. The program constraints meant that the means and standard deviations of the TAS-20 scores for the lower TAS-20 contact lists were the same for the lower and higher BIS-11 contact lists. The same applied to the higher TAS-20 contact list. Similarly, the means and standard deviations of the BIS-11 scores for the lower BIS-11 contact lists were the same for the lower and higher TAS-20 contact lists. As before, the same applied to the higher BIS-11 contact list. A final constraint was that an equal numbers of males and females were included in each group. These 80 participants were then contacted and asked to participate in the experiment. They were told that there were monetary prizes of £25, £15, and £10 to be awarded for the three highest scores.

A total of 49 participants replied. The cell sizes were 11, 12, 13, and 13 for the four groups of low/high TAS-20 by low/high BIS-11. The number of males and females in each group was not significantly different, with a total of 27 females and 22 males participating. The average age of the participants was 21.1 years. Overall (see Table 4), for the TAS-20 scores there was a significant difference between low and high TAS-20 scorers on the TAS-20 but not on the BIS-11. Similarly, there was a significant difference between the low and high BIS-11 scorers on the BIS-11 but no difference for these groups on the TAS-20. No other significant differences were found. For the high TAS-20 group of participants all the participants met the criteria for at or near caseness (Taylor et al., 1997).

**Table 4:** **Means (and standard errors) of the TAS-20 and BIS-11 scores for the alexithymia by impulsivity groups**.

		**TAS-20 scores**	**BIS-11 scores**
Low TAS-20	Low BIS-11 (*N* = 11)	40.46 (2.21)	62.36 (2.02)
	High BIS-11 (*N* = 13)	42.92 (2.04)	81.00 (1.86)
High TAS-20	Low BIS-11 (*N* = 13)	62.39 (2.04)	60.92 (1.86)
	High BIS-11 (*N* = 12)	57.42 (2.12)	84.92 (1.93)

#### Toronto alexithymia scale (TAS-20)

The details are the same as in Experiment 1.

#### Barratt impulsiveness scale

The Barratt Impulsiveness Scale, version 11 (BIS-11; Patton et al., 1995) is a 30 item self-report questionnaire assessing impulsiveness. It is constructed on the basis of six first order impulsiveness factors (attention—“I don't ‘pay attention,”’ cognitive instability—“I often have extraneous thoughts when thinking,” motor—“I do things without thinking,” perseverance—“I can only think about one thing at a time,” self-control—“I plan task carefully” and cognitive complexity—“I like puzzles”). The items are scored on a scale from 1 (Rarely/Never) to 4 (Almost Always/Always) with a higher total score associated with higher impulsivity (once reversed items are accounted for).

#### Cambridge gambling task

The procedure was the same as in Experiment 1 with one exception. In the first experiment the amount available to bet increased in 10% steps (one step per second). In this experiment, the amount available to bet decreases in 10% steps from 90%.

This study was carried out in accordance with the recommendations of the School of Psychology, University of Nottingham Ethics committee with written informed consent from all participants. All participants gave written informed consent in accordance with the Declaration of Helsinki.

### Results

As in the previous experiment, the total number of points scored was significantly skewed so the data was logarithm (base 10) transformed. A 2 (alexithymia: high vs. low) × 2 (impulsivity: high vs. low) between groups analysis was then conducted on the logarithm transformed scores with the first variable being alexithymia and the second impulsivity (see Table 5 for the means and standard errors).

**Table 5:** **Means (and standard errors) of the total points scored for alexithymia by impulsivity**.

	**Lower BIS-11**	**Higher BIS-11**
Lower TAS-20	4.83 × 10^6^ (3.94 × 10^10^)	9.66 × 10^9^ (3.63 × 10^10^)
Higher TAS-20	7.06 × 10^10^ (3.63 × 10^10^)	2.51 × 10^8^ (3.78 × 10^10^)

There was no significant effect for TAS-20 group [*F*_(1, 45)_ = 0.82, *MSe* = 5.01, *p* = 0.37, $\eta_{p}^{2}$ = 0.02], nor BIS-11 group [*F*_(1, 45)_ = 2.21, *MSe* = 5.01, *p* = 0.14, $\eta_{p}^{2}$ = 0.05]. There was also no significant interaction between TAS-20 and BIS-11 groups [*F*_(1, 45)_ = 3.51, *MSe* = 5.01, *p* = 0.07, $\eta_{p}^{2}$ = 0.02].

To establish whether participants were behaving rationally with respect to the probability of winning a 2 (alexithymia) × 2 (impulsivity) × 5 (probability of winning) analysis of variance was conducted on the proportion of the available stake that was bet (see Table 6 for the means and standard errors). The only main effect that was significant was that of the probability of winning [*F*_(1.94, 87.16)_ = 64.63, *MSe* = 0.02, *p* < 0.01, $\eta_{p}^{2}$ = 0.59], with neither alexithymia [*F*_(1, 45)_ = 0.11, *MSe* = 0.18, *p* = 0.74, $\eta_{p}^{2}$ < 0.01] nor impulsivity [*F*_(1, 45)_ = 0.59, *MSe* = 0.18, *p* = 0.45, $\eta_{p}^{2}$ = 0.01] having significant effects. None of the two way interactions were significant [alexithymia × impulsivity: *F*_(1, 45)_ = 1.62, *MSe* = 0.18, *p* = 0.24, $\eta_{p}^{2}$ = 0.04; alexithymia × probability of winning: *F*_(1.94, 87.16)_ = 0.55, *MSe* = 0.02, *p* = 0.57, $\eta_{p}^{2}$ = 0.01; impulsivity × probability of winning: *F*_(1.94, 87.16)_ = 0.09, *MSe* = 0.02, *p* = 0.91, $\eta_{p}^{2}$ < 0.01]. Finally, the three-way interaction was not significant [*F*_(1.94, 87.16)_ = 0.14, *MSe* = 0.02, *p* = 0.87, $\eta_{p}^{2}$ < 0.01].

**Table 6:** **Means (and standard errors) of the proportion bet by alexithymia and impulsivity by probability of winning on the current trial**.

**Alexithymia**	**Impulsivity**	**Probability of winning**				
		***p* = 0.5**	***p* = 0.6**	***p* = 0.7**	***p* = 0.8**	***p* = 0.9**
Lower	Lower	0.54 (0.06)	0.62 (0.06)	0.68 (0.05)	0.76 (0.05)	0.82 (0.05)
	Higher	0.46 (0.06)	0.53 (0.05)	0.57 (0.05)	0.67 (0.05)	0.75 (0.04)
Higher	Lower	0.46 (0.06)	0.57 (0.05)	0.64 (0.05)	0.74 (0.05)	0.81 (0.04)
	Higher	0.50 (0.06)	0.59 (0.05)	0.66 (0.05)	0.76 (0.05)	0.80 (0.04)
	Overall	0.49 (0.03)	0.58 (0.03)	0.64 (0.026)	0.73 (0.02)	0.80 (0.02)

To test whether loss-chasing behavior was affected by alexithymia and impulsivity, a 2 (alexithymia) × 2 (impulsivity) × 2 (previous trial) × 5 (probability of winning) mixed analysis of variance was conducted on the proportion of the stake that was bet. The means and standard errors are found in Table 7.

**Table 7:** **Means (and standard errors) of the proportion bet for alexithymia and impulsivity by outcome of the previous trial and probability of winning on the current trial**.

**Alexithymia**	**Impulsivity**	**Previous trial**	**Probability of winning**				
			***p* = 0.5**	***p* = 0.6**	***p* = 0.7**	***p* = 0.8**	***p* = 0.9**
Lower	Lower	Won	0.50 (0.06)	0.60 (0.06)	0.66 (0.06)	0.73 (0.05)	0.82 (0.05)
		Lost	0.52 (0.07)	0.65 (0.05)	0.70 (0.06)	0.80 (0.05)	0.83 (0.04)
	Higher	Won	0.45 (0.06)	0.48 (0.06)	0.54 (0.05)	0.65 (0.05)	0.74 (0.04)
		Lost	0.46 (0.06)	0.65 (0.05)	0.65 (0.05)	0.72 (0.05)	0.80 (0.04)
Higher	Lower	Won	0.42 (0.06)	0.54 (0.06)	0.61 (0.05)	0.73 (0.05)	0.79 (0.04)
		Lost	0.63 (0.06)	0.72 (0.05)	0.71 (0.05)	0.78 (0.05)	0.83 (0.04)
	Higher	Won	0.44 (0.06)	0.55 (0.06)	0.62 (0.05)	0.75 (0.05)	0.79 (0.05)
		Lost	0.63 (0.06)	0.68 (0.05)	0.75 (0.06)	0.80 (0.05)	0.82 (0.04)

Both the main effects of previous trial [*F*_(1, 45)_ = 57.01, *MSe* = 0.02, *p* < 0.01, $\eta_{p}^{2}$ = 0.56] and probability of winning [*F*_(1.94, 87.16)_ = 0.14, *MSe* = 0.04, *p* < 0.01, $\eta_{p}^{2}$ = 0.59] were significant but neither alexithymia [*F*_(1, 45)_ = 0.60, *MSe* = 0.20, *p* = 0.44, $\eta_{p}^{2}$ = 0.01] nor impulsivity [*F*_(1, 45)_ = 0.61, *MSe* = 0.20, *p* = 0.44, $\eta_{p}^{2}$ = 0.01] were significant. Overall, participants bet a larger proportion of the available stake after a loss (mean = 0.71) than a win (mean = 0.62). Furthermore, as the probability of winning increased participants bet more (*p*_0.5_ = 0.51, *p*_0.6_ = 0.61, *p*_0.7_ = 0.65, *p*_0.8_ = 0.75, *p*_0.9_ = 0.80).

**Figure 3:**
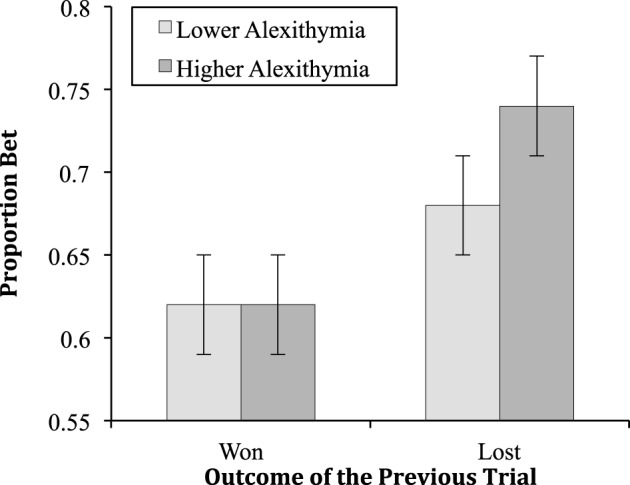
**The means (and standard errors) of the proportion bet for the alexithymia groups having won or lost on the previous trial**.

**Figure 4:**
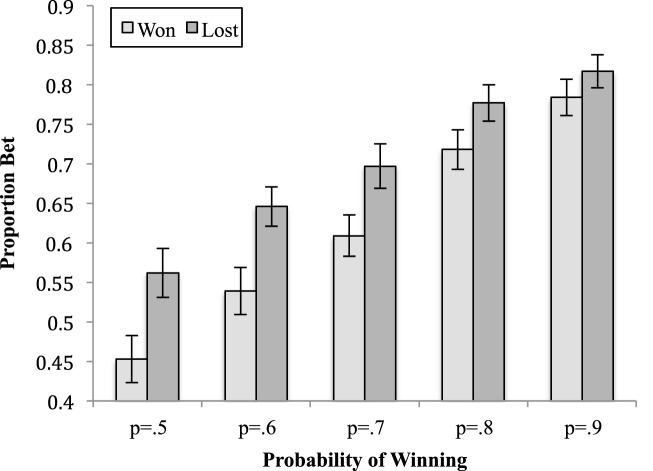
**The means (and standard errors) of the proportion bet for the outcome of the previous trial by the probability of winning on the current trial**.

The two-way interaction between alexithymia and previous trial [*F*_(1, 45)_ = 4.54, *MSe* = 0.02, *p* = 0.04, $\eta_{p}^{2}$ = 0.09; see Figure 3] was significant; as was the interaction between previous trial and the probability of winning [*F*_(2.89, 130.06)_ = 3.85, *MSe* = 0.01, *p* = 0.01, $\eta_{p}^{2}$ = 0.08; see Figure 4]. The interactions between alexithymia and impulsivity [*F*_(1, 45)_ = 0.81, *MSe* = 0.20, *p* = 0.37, $\eta_{p}^{2}$ = 0.02], alexithymia and probability of winning [*F*_(2.30, 103.40)_ = 0.22, *MSe* = 0.04, *p* = 0.93, $\eta_{p}^{2}$ < 0.01], impulsivity and previous trial [*F*_(1, 45)_ = 0.50, *MSe* = 0.02, *p* = 0.48, $\eta_{p}^{2}$ = 0.01], and impulsivity and probability winning [*F*_(2.30, 103.40)_ = 0.04, *MSe* = 0.04, *p* = 0.99, $\eta_{p}^{2}$ < 0.01] were not significant.

The simple effects for the two-way interaction between alexithymia and previous trial showed that there was no difference between the lower and higher alexithymia groups when the previous trial was won (*p* = 0.87) but there was when the previous trial was lost (*p* = 0.02). In this latter case, participants higher in alexithymia bet proportionally more after a loss than those lower in alexithymia. For the lower alexithymia group there was a significant difference between having won or lost on the previous trial (*p* < 0.01) as there was for the higher alexithymia group (*p* < 0.01). In both cases, both the lower and higher alexithymia participants bet proportionally more after a loss than after a win.

For the two-way interaction between previous trial and probability of winning, the simple effect show that there was a significant different between when the previous trial was won or lost at each level of level probability of winning on the current trial (*p*_min_ = 0.02, *p*_max_ < 0.01). In each case, participants bet significantly more after a loss than a win with the difference between the two decreasing as the probability of winning on the current trial increased.

**Figure 5:**
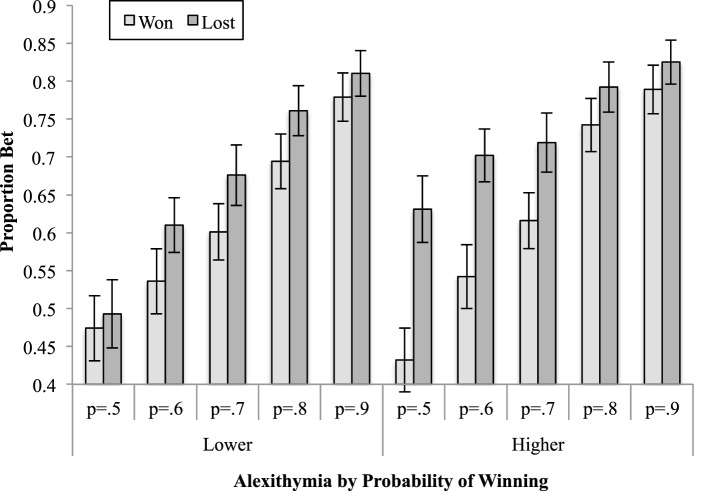
**The means (and standard errors) of the proportion bet for the outcome of the previous trial by the probability of winning on the current trial**.

The three-way interaction between alexithymia, previous trial, and probability of winning [*F*_(2.89, 130.60)_ = 3.50, *MSe* = 0.01, *p* = 0.01, $\eta_{p}^{2}$ = 0.07; see Figure 5] was significant but the interactions between alexithymia, impulsivity, and previous trial [*F*_(1, 45)_ = 2.07, *MSe* = 0.02, *p* = 0.16, $\eta_{p}^{2}$ = 0.04] and between impulsivity, previous trial and probability of winning [*F*_(2.89, 130.60)_ = 0.24, *MSe* = 0.01, *p* = 0.86, $\eta_{p}^{2}\;$ < 0.01] were not significant. Neither was the four-way interaction between alexithymia, impulsivity, previous trial, and probability of winning [*F*_(2.89, 130.60)_ = 0.70, *MSe* = 0.01, *p* = 0.55, $\eta_{p}^{2}$ = 0.02].

The three-way interaction between alexithymia, previous trial and probability of winning was further analyzed by examining the two-way interaction between previous trial and probability of winning separately for the lower and higher alexithymia participants. For the lower alexithymia group this interaction was not significant [*F*_(2.48, 54.51)_ = 1.39, *MSe* = 0.02, *p* = 0.26, $\eta_{p}^{2}$ < 0.06], although both the main effects of previous trial and probability of winning were significant. On the other hand, it was significant for the higher alexithymia group [*F*_(2.73, 62.70)_ = 7.04, *MSe* = 0.01, *p* < 0.01, $\eta_{p}^{2}$ = 0.23]. A linear contrast for the latter interaction was significant [*F*_(1, 23)_ = 14.02, *MSe* = 0.02, *p* < 0.01, $\eta_{p}^{2}$ = 0.37], indicating that the gradient of the trend, for the proportion bet as the probability of winning rises to increase, is different when the previous trial was won rather than lost. As can be seen in Figure 5, when the previous trial was won the proportion bet ranges from 0.43 (probability of winning is 0.5) to 0.79 (probability of winning is 0.9), whereas it ranges from 0.63 (probability of winning is 0.5) to 0.83 (probability of winning is 0.9) when the previous trial was lost. This represents a ceiling effect for both groups (since the maximum available bet was 90% of the available stake) which is reached at lower probabilities of winning for the high alexithymia group.

Finally, to check that there was no confounding effect of sex of participant all the analyses conducted so far for experiment 2 were conducted again this time replacing the impulsivity variable with the sex of participant variable. For total points scored, proportion bet on a trial for the current trial and proportion bet on a trial after a previous win or loss there were no significant main effects of sex or interactions between sex and the other variables. At the same time, all the previously significant effects remained statistically significant. Similarly, the previously non-significant effects remained non-significant.

To explore which facet of alexithymia contributes most to the difference in proportion bet depending on whether the previous trial was won or lost, a difference score was calculated between the proportion bet after a loss and the proportion bet after a win for the five levels of the probability of winning. These difference scores were then correlated with the three facets of the TAS-20 alexithymia scale (see Table 8). Both DIF and difficulty describing feeling (DDF) were positively correlated with the lost/won difference score when the probability of winning was 0.5, and DIF was also significantly positively correlated when the probability of winning was 0.6. EOT was not correlated with any of the difference scores. It is possible that as the probability of winning increases that any underlying correlation between alexithymia and the difference scores is obscured since the variability in the difference scores is simultaneously decreasing.

**Table 8:** **Correlations (means and standard errors) between the lost/won difference scores at each level of probability of winning and the three facets of alexithymia**.

**Probability of winning**	**TAS-20 factors**			**Mean (S.E.)**
	**DIF**	**DDF**	**EOT**	
*p* = 0.5	0.34^*^	0.39^**^	0.18	0.14 (0.034)
*p* = 0.6	0.29^*^	0.17	0.17	0.11 (0.024)
*p* = 0.7	0.08	0.10	0.03	0.09 (0.017)
*p* = 0.8	0.11	0.07	−0.06	0.06 (0.014)
*p* = 0.9	−0.00	−0.03	−0.07	0.03 (0.14)
Mean (S.E.)	15.20	13.14	22.86	

* *p < 0.05*,

** *p < 0.01. DIF, Difficulty Identifying Feelings; DDF, Difficulty Describing Feelings; EOT, Externally Orienting Thinking*.

### Discussion

By adapting the gambling task so that participants had to wait for a shorter time to place larger bets the overall proportion bet was substantially greater than in Experiment 1. Overall, in Experiment 1 participants bet 24.0% (of the available stake) but in Experiment 2 this increased to 64.4%. Participants bet more than 2.5 times as much in Experiment 2 as in Experiment 1. All four groups of participants demonstrated a linear increase in the mean proportion bet as the probability of winning increased. As in Experiment 1 this suggests that all the participants understood the task and behaved accordingly.

With respect to alexithymia and previous wins or losses, the predicted interaction was observed. First, participants low in alexithymia demonstrated loss-chasing betting more after a loss (67.8%) than a win (61.7%; *d* = 0.43). At least part of loss-chasing in this task reflects something other than alexithymia. It is likely, that given the goal of maximizing the points won, low alexithymia participants realized that a large loss needed to be compensated and thus they gambled more than they might otherwise. The participants who were high in alexithymia also chased their losses; after a win they bet 62.4% but after a loss 73.4% (*d* = 0.79). The size of effect has increased by a factor of 3 in comparison to Experiment 1 and is approximately twice as large for the high alexithymia as for the low alexithymia participants.

The three-way interaction between alexithymia, previous trial, and probability of winning can be interpreted as a ceiling effect that operates particular strongly for the high alexithymia participants. For the low alexithymia participants the loss-chasing effect was approximately the same size for all probabilities of winning, approximately, 5.3%. However, for the high alexithymia participants the loss-chasing effect diminishes from 19.9% when the probability of winning was 0.5–3.6% when the probability of winning was 0.9. This seems counterintuitive given that usually it is a good idea to bet more as the likelihood of winning increases. However, it is likely that this simply reflects an artifactual ceiling effect. On average the maximum bet that could be made was 90% of the available stake and after a loss participants high in alexithymia bet on average 82.5%. It was not actually possible for them to bet more than 100% of the available stake which would be necessary for them to have a loss-chasing effect the same size as when the probability of winning was 0.5.

The final alexithymia effects are the correlations between the facets of alexithymia, DIF, DDF, and externally oriented thought. Both DIF and DDF showed a significant positive correlation with the average loss-chasing effect (i.e., proportion bet after a loss—proportion bet after a win) but the EOT facet was not significant. This suggests that it is the emotional and not the cognitive component of alexithymia that is related to loss-chasing.

In terms of overall performance, that impulsivity showed no effect on either the average amount bet or the average bet after a winning or losing trial is striking. The predicted interaction between alexithymia and impulsivity was not statistically significant. One possible explanation for this failure to find any impulsivity effects is that the participants selected were not really impulsive. In a recent re-evaluation of the BIS-11 scale Stanford et al. (2009) report a sample of 1577 adults who completed the BIS-11. The mean BIS-11 score for these individuals was 62.3 (*SD* = 10.3). Both the lower and higher TAS-20, lower impulsivity groups' 95 and 99% confidence intervals for the mean include this mean, indicating that these groups are scoring at reasonably near the population mean. Neither of the lower and higher TAS-20, higher impulsivity groups' 95 and 99% confidence intervals for the mean include 62.3. Of the 25 participants in the high impulsivity groups all would be in the top 20% of Stanford et al.'s (2009) sample, 21 would be in the top 10% and 13 in the top 5%. We can be reasonably certain that these latter groups are on average significantly more impulsive than the general population. It seems unlikely that not being impulsive is the issue.

A second possibility is that the waiting constraint (i.e., participants had to wait as the bet value stepped down in 1 s intervals) overcame any impulsivity effect. Since on average participants in this second experiment bet 64% of the available stake they were forced to wait on average 3.5 s before they could select their bet. The more impulsive participants had to wait to place a rational bet. Future studies looking at impulsivity in this task could choose to lift this constraint by using a different bet input method. For example, using a slider on the screen to select the level of the bet the participants would wish to place would allow the more impulsive participants to place a bet more quickly.

## General discussion

In both experiments a loss-chasing effect was observed for participants who scored higher in alexithymia. It was hypothesized that this would be the case since alexithymic individuals are less sensitive to losses (Ferguson et al., 2009; Bibby and Ferguson, 2011). A comparison of the size of the loss-chasing effect for the alexithymics between the two experiments suggests that it is proportional to the size of the loss. The loss-chasing effect is small when the loss is small and larger when the loss is larger. This is a new and interesting observation. For non-alexithymic individuals when the loss was small there was no evidence of loss-chasing but when the loss was relatively large a loss-chasing effect emerged. However, the comparison between alexithymics and non-alexithymics indicated that the loss-chasing effect was substantially greater for the alexithymics.

Two possible explanations for why loss-chasing occurs involve the cognitive demands associated with recouping losses and the failure of the affect processing systems to successfully process the emotional consequences of losses. The current experiments suggest that both these factors are in operation. For both alexithymic and non-alexithymic participants a financial reward was only obtained if they scored sufficiently high on the gambling task. If a substantial loss was made, and in the second experiment it was, then given that the task was time limited (a fact that was made clear to all the participants), then the need to maximize the overall score was important if the monetary reward was to be achieved. Participants were under greater pressure to recover from losses in experiment 2, given that the average percentage bet was 24.0% in experiment 1 and 64.4% in experiment 2 and therefore the losses were greater. Under such conditions betting more after a loss is not lacking in rationale. It is a gamble that could pay off.

Utilizing how losses make you feel is also important. A loss feels bad. As Kahneman and Tversky (Kahneman and Tversky, 1979; Tversky and Kahneman, 1981) have pointed out, people are so intolerant of losses that on average they will not gamble if there is the possibility of a loss unless the potential gain is approximately twice the size of the potential loss. However, if the associated emotional experience of a loss is missing, misinterpreted or ignored then this bias against making losses is less likely to be experienced. Taylor et al. (1997) suggest that alexithymia is not the same as athymia. It is not that alexithymics do not experience emotions, rather it is the sense making and processes of affect regulation associated with emotions that is dysfunctional in alexithymia. In the current task, the negative emotions associated with a loss are likely to be misinterpreted or ignored by those high in alexithymia. If this is the case, then the cognitive to demand to recoup losses will outweigh the emotional demand to minimize them leading to loss-chasing which is further exacerbated by the size of the loss. That it is a deficit in emotion processing that is the problem is supported by the positive correlations between the emotional facets of alexithymia, DIF and DDF, and loss-chasing and the failure to find a correlation between the cognitive component of alexithymia, EOT.

With respect to gambling, the literature is relatively clear. Loss-chasing is a key feature of problem gambling. As Lesieur (1979) has pointed out it is loss-chasing that gets the problem gambler in trouble in the first place. Having lost money, the problem gambler bets more to try to win what they have lost. However, they overbet, leading them to lose more money. Then they chase this. It should be noted that the loss-chasing Lesieur discusses is at a grander scale than the loss-chasing examined in the current research. However, the similarities between within session and between session loss-chasing should not be ignored (Breen and Zuckerman, 1999). Furthermore, as Breen and Zuckerman (1999) argue within session loss chasing may well lead to between session loss chasing. At the same time alexithymia is a common feature of problem gamblers. While not all problem gamblers are alexithymic, alexithymia is more highly represented in this population than in the normal population. The findings reported here suggest that it is not a coincidence that problem gambling and alexithymia coexist. Alexithymics are more likely to loss-chase which the evidence suggests is more likely to lead to problem gambling.

The neurological studies reported earlier support the idea that reduced activity in the emotion centers of the brain is associated with both alexithymia and loss-chasing. In particular, Campbell-Meiklejohn et al. (2008) suggest that less activity in the regions associated with managing conflict between cognitive and emotional systems could be a precursor to loss-chasing. It is these, and other, regions that been found to show reduced activity in alexithymics. Damasio and colleagues (Damasio, 1996; Bechara, 1999; Bechara et al., 2005) have demonstrated that injury to the emotion processing centers of the brain, in particular the prefrontal cortex, lead to poor decision making in the IGT. The loss-chasing effect for alexithymics is another example of how failing to utilize emotional information can lead to poor decision making.

The current studies only considered two personality variables, alexithymia and impulsivity when trying to explain loss-chasing behavior. No doubt there are other personality variables that are important (e.g., Kim and Lee, 2011). Future studies should consider including a more complete battery of personality measures to establish whether alexithymia acts independently of such personality variables or is either moderated or mediated by them. Furthermore, at least part of the justification for conducting these studies was the relationships between alexithymia and problem gambling and between problem gambling and loss-chasing. The current studies were conducted with undergraduate students as participants. They were not screened for problem gambling. Thus, an important extension of this work would be to look at problem gamblers. It can be hypothesized that a specific subgroup of problem gamblers, those high in alexithymia, are likely to loss-chase more. It may even be the case that problem gamblers low in alexithymia do not loss-chase within a gambling session. If they then loss-chase between gambling sessions it seems likely that this would be explained by a different mechanism.

A further limitation of the study is that it does not specifically address the exact mechanism by which a general deficit in processing emotional information affects participants' responses to losses but does not affect participants' responses to wins. Previous research (Ferguson et al., 2009; Bibby and Ferguson, 2011) has found a relationship between alexithymia and deficits in processing losses. However, it is not currently known why this should happen. However, the earliest descriptions of alexithymia specifically identified reduced awareness of negative feelings and emotions as a problem and not positive emotions. Lane et al. (2000) found a correlation between negative affect and alexithymia but not positive affect. This could be because that there is a smaller number of positive rather than negative emotions typically expressed so it is easier to learn about positive affect than negative affect when there is a general deficit in processing emotions. As Taylor et al. (1997) have argued it is specifically the regulation of the emotions, the making sense of them, which is important in alexithymia.

Ferguson et al. (2009) suggested that further studies of decision making in alexithymia should examine other individual differences. In particular, given that the IGT, and in this case the CGT, involve risky decision making impulsivity should be considered. Experiment 1 demonstrated that alexithymia was associated with loss-chasing and experiment 2 incorporated impulsivity as possible contributor to loss-chasing. The results, however, suggest that impulsivity is not directly related to loss-chasing. Breen and Zuckerman (1999) reported a similar failure to find an association between impulsivity and loss-chasing. This is not to suggest that impulsivity is not associated with problem gambling, rather, it is not associated with one aspect of problem gambling, that is, loss-chasing.

## Author contributions

The author received assistance in collecting the data but is otherwise responsible for the design, implementation, statisitcal analysis, and reporting of this research.

The reviewer, Arianna Palmieri, and handling Editor, Antonino Vallesi declared their shared affiliation, and the handling Editor states that the process nevertheless met the standards of a fair and objective review.

### Conflict of interest statement

The author declares that the research was conducted in the absence of any commercial or financial relationships that could be construed as a potential conflict of interest. The reviewer, Arianna Palmieri, and handling Editor declared their shared affiliation, and the handling Editor states that the process nevertheless met the standards of a fair and objective review.

## Footnotes

^1^Throughout the paper, *F* statistics including this variable were Greenhouse and Geisser corrected to take account of the failure to meet the sphericity assumption.

## References

[B1] Alessi S. M. Petry N. M. (2003). Pathological gambling severity is associated with impulsivity in a delay discounting procedure. Behav. Processes 64, 345–354. 10.1016/S0376-6357(03)00150-5 14580703

[B2] Bagby R. M. Parker J. D. Taylor G. J. (1994). The 20-item Toronto-Alexithymia-Scale 1: item selection and cross-validation of the factor structure. J. Psychosom. Res. 38, 23–32. 10.1016/0022-3999(94)90005-1 8126686

[B3] Bagby R. M. Quilty L. C. Taylor G. J. Grabe H. J. Luminet O. Verissimo R. (2009). Are there subtypes of alexithymia? Pers. Individ. Dif. 47, 413–418. 10.1016/j.paid.2009.04.012

[B4] Bagby R. M. Taylor G. J. Quilty L. C. Parker J. D. A. (2007). Reexamining the factor structure of the 20-item Toronto Alexithymia Scale: commentary on Gignac, Palmer, and Stough. J. Pers. Assess. 89, 258–264. 10.1080/00223890701629771 18001226

[B5] Bechara A. (1999). Emotion and decision-making after frontal lobe damage: implications for understanding certain disorders from psychiatry. Nervenheilkunde 18, 54–59.

[B6] Bechara A. Damasio A. R. Damasio H. Anderson S. W. (1994). Insensitivity to future consequence following damage to human pre-frontal cortex. Cognition 50, 7–15. 10.1016/0010-0277(94)90018-3 8039375

[B7] Bechara A. Damasio H. Tranel D. Damasio A. R. (2005). The Iowa Gambling Task and the somatic marker hypothesis: some questions and answers. Trends Cogn. Sci. 9, 159–162. 10.1016/j.tics.2005.02.002 15808493

[B8] Berthoz S. Artiges E. Van De Moortele P. F. Poline J. B. Rouquette S. Consoli S. M.. (2002). Effect of impaired recognition and expression of emotions on frontocingulate cortices: an fMRI study of men with alexithymia. Am. J. Psychiatry 159, 961–967. 10.1176/appi.ajp.159.6.961 12042184

[B9] Bibby P. A. Ferguson E. (2011). The ability to process emotional information predicts loss aversion. Pers. Individ. Dif. 51, 263–266. 10.1016/j.paid.2010.05.001

[B10] Blaszczynski A. Steel Z. McConaghy N. (1997). Impulsivity in pathological gambling: the antisocial impulsivist. Addiction 92, 75–87. 10.1111/j.1360-0443.1997.tb03639.x 9060199

[B11] Bonnaire C. Bungener C. Varescon I. (2009). Subtypes of French pathological gamblers: comparison of sensation seeking, alexithymia and depression scores. J. Gambl. Stud. 25, 455–471. 10.1007/s10899-009-9142-z 19636683

[B12] Breen R. B. Zuckerman M. (1999). ‘Chasing’ in gambling behavior: personality and cognitive determinants. Pers. Individ. Dif. 27, 1097–1111. 10.1016/S0191-8869(99)00052-5

[B13] Campbell-Meiklejohn D. K. Woolrich M. W. Passingham R. E. Rogers R. D. (2008). Knowing when to stop: the brain mechanisms of chasing losses. Biol. Psychiatry 63, 293–300. 10.1016/j.biopsych.2007.05.014 17662257

[B14] Corless T. Dickerson M. (1989). Gamblers' self-perceptions of the determinants of impaired control. Br. J. Addict. 84, 1527–1537. 10.1111/j.1360-0443.1989.tb03936.x 2611437

[B15] Damasio A. R. (1996). The somatic marker hypothesis and the possible functions of the prefrontal cortex. Philos. Trans. R. Soc. B Biol. Sci. 351, 1413–1420. 10.1098/rstb.1996.0125 8941953

[B16] Dickerson M. Hinchy J. Fabre J. (1987). Chasing, Arousal and sensation seeking in off-course gamblers. Br. J. Addict. 82, 673–680. 10.1111/j.1360-0443.1987.tb01530.x 3475105

[B17] Ferguson E. Bibby P. A. Rosamond S. O'Grady C. Parcell A. Amos C.. (2009). Alexithymia, cumulative feedback, and differential response patterns on the iowa gambling task. J. Pers. 77, 883–902. 10.1111/j.1467-6494.2009.00568.x 20078741

[B18] Gray J. A. McNaughton N. (2000). The Neuropsychology of Anxiety: An Enquiry into the Functions of the Septo-Hippocampal System. Oxford: Oxford University Press.

[B19] Gunnarsson M. Gustavsson J. P. Tengstrom A. Franck J. Fahlke C. (2008). Personality traits and their associations with substance use among adolescents. Pers. Individ. Dif. 45, 356–360. 10.1016/j.paid.2008.05.004

[B20] Gustavsson J. P. Jonsson E. G. Linder J. Weinryb R. M. (2003). The HP5 inventory: definition and assessment of five health-relevant personality traits from a five-factor model perspective. Pers. Individ. Dif. 35, 69–89. 10.1016/S0191-8869(02)00142-3

[B21] Kahneman D. Knetsch J. L. Thaler R. H. (1990). Experimental tests of the endowment effect and the COASE theorem. J. Polit. Econ. 98, 1325–1348. 10.1086/261737

[B22] Kahneman D. Tversky A. (1979). Prospect theory - Analysis of decision under risk. Econometrica 47, 263–291. 10.2307/1914185

[B23] Kano M. Fukudo S. Gyoba J. Kamachi M. Tagawa M. Mochizuki H.. (2003). Specific brain processing of facial expressions in people with alexithymia: an (H2O)-O-15-PET study. Brain 126, 1474–1484. 10.1093/brain/awg131 12764066

[B24] Kano M. Hamaguchi T. Itoh M. Yanai K. Fukudo S. (2007). Correlation between alexithymia and hypersensitivity to visceral stimulation in human. Pain 132, 252–263. 10.1016/j.pain.2007.01.032 17360119

[B25] Kim D. Y. Lee J. H. (2011). Effects of the BAS and BIS on decision-making in a gambling task. Pers. Individ. Dif. 50, 1131–1135. 10.1016/j.paid.2011.01.041

[B26] Kugel H. Eichmann M. Dannlowski U. Ohrmann P. Bauer J. Arolt V.. (2008). Alexithymic features and automatic amygdala reactivity to facial emotion. Neurosci. Lett. 435, 40–44. 10.1016/j.neulet.2008.02.005 18314269

[B27] Lane R. D. Reiman E. M. Axelrod B. Yun L. S. Holmes A. Schwartz G. E. (1998). Neural correlates of levels of emotional awareness. Evidence of an interaction between emotion and attention in the anterior cingulate cortex. J. Cogn. Neurosci. 10, 525–535. 10.1162/089892998562924 9712681

[B28] Lane R. D. Sechrest L. Riedel R. Shapiro D. E. Kaszniak A. W. (2000). Pervasive emotion recognition deficit common to alexithymia and repressive coping style. Psychosom. Med. 62, 492–501. 10.1097/00006842-200007000-00007 10949094

[B29] Lesieur H. R. (1979). Compulsive Gambler's spiral of options and involvement. Psychiatry Interpers. Biol. Process. 42, 79–87. 760136 10.1080/00332747.1979.11024008

[B30] Lesieur H. R. Blume S. B. (1987). The South Oak Gambling Screen (SOGS) – a new instrument for the identification of pathological gamblers. Am. J. Psychiatry 144, 1184–1188. 10.1176/ajp.144.9.1184 3631315

[B31] Lin C.-H. Chiu Y.-C. Lee P.-L. Hsieh J.-C. (2007). Is deck B a disadvantageous deck in the Iowa Gambling Task? Behav. Brain Funct. 3, 1–10. 10.1186/1744-9081-3-16 17362508 PMC1839101

[B32] Linnet J. Røjskjaer S. Nygaard J. Maher B. A. (2006). Episodic chasing in pathological gamblers using the Iowa gambling task. Scand. J. Psychol. 47, 43–49. 10.1111/j.1467-9450.2006.00491.x 16433661

[B33] Lumley M. A. Roby K. J. (1995). Alexithymia and Pathological gambling. Psychother. Psychosom. 63, 201–206. 10.1159/000288960 7624467

[B34] MacLaren V. V. Fugelsang J. A. Harrigan K. A. Dixon M. J. (2011). The personality of pathological gamblers: a meta-analysis. Clin. Psychol. Rev. 31, 1057–1067. 10.1016/j.cpr.2011.02.002 21802620

[B35] Mantani T. Okamoto Y. Shirao N. Okada G. Yamawaki S. (2005). Reduced activation of posterior cingulate cortex during imagery in subjects with high degrees of alexithymia: a functional magnetic resonance imaging study. Biol. Psychiatry 57, 982–990. 10.1016/j.biopsych.2005.01.047 15860338

[B36] Mitrovic D. V. Brown J. (2009). Poker mania and problem gambling: a study of distorted cognitions, motivation and alexithymia. J. Gambl. Stud. 25, 489–502. 10.1007/s10899-009-9140-1 19649568

[B37] Moriguchi Y. Ohnishi T. Lane R. D. Maeda M. Mori T. Nemoto K.. (2006). Impaired self-awareness and theory of mind: an fMRI study of mentalizing in alexithymia. Neuroimage 32, 1472–1482. 10.1016/j.neuroimage.2006.04.186 16798016

[B38] O'Connor J. Dickerson M. (2003). Impaired control over gambling in gaming machine and off-course gamblers. Addiction 98, 53–60. 10.1046/j.1360-0443.2003.00232.x 12492755

[B39] Parker J. D. A. Bagby R. M. Taylor G. J. Endler N. S. Schmitz P. (1993). Factorial validity of the 20-item Toronto alexithymia scale. Eur. J. Pers. 7, 221–232. 10.1002/per.2410070403

[B40] Parker J. D. A. Keefer K. V. Taylor G. J. Bagby R. M. (2008). Latent structure of the alexithymia construct: a taxometric investigation. Psychol. Assess. 20, 385–396. 10.1037/a0014262 19086762

[B41] Parker J. D. A. Wood L. M. Bond B. J. Shaughnessy P. (2005). Alexithymia in young adulthood: a risk factor for pathological gambling. Psychother. Psychosom. 74, 51–55. 10.1159/000082027 15627857

[B42] Patton J. H. Stanford M. S. Barratt E. S. (1995). Factor structure of the Barratt impulsive scale. J. Clin. Psychol. 6, 768–774. 8778124 10.1002/1097-4679(199511)51:6<768::aid-jclp2270510607>3.0.co;2-1

[B43] Rogers R. D. Everitt B. J. Baldacchino A. Blackshaw A. J. Swainson R. Wynne K.. (1999). Dissociable deficits in the decision-making cognition of chronic amphetamine abusers, opiate abusers, patients with focal damage to prefrontal cortex, and tryptophan-depleted normal volunteers: evidence for monoaminergic mechanisms. Neuropsychopharmacology 20, 322–339. 10.1016/S0893-133X(98)00091-8 10088133

[B44] Shishido H. Gaher R. M. Simons J. S. (2013). I don't know how I feel, therefore I act: alexithymia, urgency, and alcohol problems. Addict. Behav. 38, 2014–2017. 10.1016/j.addbeh.2012.12.014 23384454

[B45] Stanford M. S. Mathias C. W. Dougherty D. M. Lake S. L. Anderson N. E. Patton J. H. (2009). Fifty years of the barratt impulsiveness scale: an update and review. Pers. Individ. Dif. 47, 385–395. 10.1016/j.paid.2009.04.008

[B46] Steel Z. Blaszczynski A. (1998). Impulsivity, personality disorders and pathological gambling severity. Addiction 93, 895–905. 10.1046/j.1360-0443.1998.93689511.x 9744125

[B47] Taylor G. J. Bagby R. M. Parker J. D. A. (1997). Disorders of Affect Regulation: Alexithymia in Medical and Psychiatric Illness. Cambridge: Cambridge University Press 10.1017/CBO9780511526831

[B48] Taylor G. J. Ryan D. Bagby R. M. (1985). Toward the development of a new self-report alexithymia scale. Psychother. Psychosom. 44, 191–199. 10.1159/000287912 3837277

[B49] Toce-Gerstein M. Gerstein D. R. Volberg R. A. (2003). A hierarchy of gambling disorders in the community. Addiction 98, 1661–1672. 10.1111/j.1360-0443.2003.00545.x 14651495

[B50] Toplak M. E. Liu E. MacPherson R. Toneatto T. Stanovich K. E. (2007). The reasoning skills and thinking dispositions of problem gamblers: a dual-process taxonomy. J. Behav. Decis. Mak. 20, 103–124. 10.1002/bdm.544

[B51] Tversky A. Kahneman D. (1981). The framing of decisons and the psychology of choice. Science 211, 453–458. 10.1126/science.7455683 7455683

[B52] Vitaro F. Arseneault L. Tremblay R. E. (1999). Impulsivity predicts problem gambling in low SES adolescent males. Addiction 94, 565–575. 10.1046/j.1360-0443.1999.94456511.x 10605852

[B53] Vitaro F. Wanner B. Ladouceur R. Brendgen M. Tremblay R. E. (2004). Trajectories of gambling during adolescence. J. Gambl. Stud. 20, 47–69. 10.1023/B:JOGS.0000016703.84727.d3 14973397

[B54] Wardle H. Moody A. Spence S. Orford J. Volberg R. Jotangia D. (2011). British Gambling Prevalence Survey 2010, 188 National Centre for Social Research.

[B55] Wickens C. M. Toplak M. E. Wiesenthal D. L. (2008). Cognitive failures as predictors of driving errors, lapses, and violations. Accident Anal. Prev. 40, 1223–1233. 10.1016/j.aap.2008.01.006 18460392

[B56] Zimmermann G. Rossier J. de Stadelhofen F. M. Gaillard F. (2005). Alexithymia assessment and relations with dimensions of personality. Eur. J. Psychol. Assess. 21, 23–33. 10.1027/1015-5759.21.1.23

